# Whole-genome sequencing of *Macrophomina phaseolina* Karbala-1 isolated from okra root rot

**DOI:** 10.1128/mra.00294-26

**Published:** 2026-06-22

**Authors:** Leith Hassan Kriedy, Rajaa G. Abdalmoohsin, Yasir N. H. Alhamiri, Adnan A. Lahuf

**Affiliations:** 1 Plant Protection Department, Agriculture College, University of Kerbala 125663, Karbala, Iraq; University of California Riverside, Riverside, California, USA

**Keywords:** *Macrophomina phaseolina*, draft genome, root rot, pathogenic isolate, Karbala, Iraq

## Abstract

In the current study, we reported the whole-genome sequence of *M. phaseolina* isolate Karbala-1, obtained from okra with root rot in Karbala Province, Iraq. Its genome is 52,342,473 bp with 4,984 (>1,000) scaffolds and 15,778 protein-coding genes. This genome is a valuable resource for studying the genetic basis of *M. phaseolina*.

## ANNOUNCEMENT

Root and seedling diseases represent an increasing threat to crop productivity and plant health worldwide (1, 2). Among these pathogens, *Macrophomina phaseolina* is a widely distributed soil-borne fungus with a broad host range, infecting more than 500 plant species belonging to over 100 botanical families (3). Recent advances in whole-genome sequencing of plant-associated fungi have significantly improved our understanding of pathogen evolution, host adaptation, and virulence mechanisms, providing valuable genomic resources for plant disease management and epidemiological studies (4, 5).

In 2025, *Macrophomina phaseolina* isolate Karbala-1 was obtained from roots of okra (*Abelmoschus esculentus* L.) plants showing browning and wilting symptoms associated with root rot disease. The samples were collected from agricultural fields in Karbala Province, Iraq (43°49′11″ E, 32°30′30″ N). Root tissues were surface-sterilized, plated on potato dextrose agar (PDA), and incubated at 25 ± 2°C for fungal isolation. Emerging colonies were subcultured to obtain a pure isolate (6). Pathogenicity of the isolate was confirmed through inoculation assays on okra seedlings and fulfillment of Koch’s postulates prior to genome sequencing.

The fungal isolate was initially identified based on morphological characteristics, including colony morphology, microsclerotia formation, and hyphal structure. Molecular confirmation of the species identity was subsequently performed using internal transcribed spacer (ITS) region sequencing amplified with ITS1/ITS4 primers (7). The resulting sequences showed greater than 99% similarity to reference sequences of *M. phaseolina* deposited in the NCBI database with accession number PZ118330.1. Additional BLASTn analysis (8) against the NCBI non-redundant nucleotide (nt) database further confirmed the taxonomic identity, revealing approximately >99% sequence similarity with previously reported *M. phaseolina* isolates.

Genomic DNA was extracted from a 7-day-old pure culture grown on PDA following previously described protocols (9). The extracted DNA was submitted to BGI (Hong Kong, China) for whole-genome sequencing. Sequencing libraries were constructed using the MGIEasy Universal DNA Library Prep Set (MGI). The prepared libraries were sequenced on the Illumina HiSeq platform, generating 150-bp paired-end reads.

A total of 3,563,358 raw reads were generated from the sequencing run. The raw reads were processed using SOAPnuke version 2.X to remove adapter sequences, low-quality bases, short reads, and potential PCR duplicates (10). After quality filtering, a total of 1,069,007,400 clean bases were retained. The sequencing data exhibited high quality, with Q20 and Q30 values of 98.04% and 93.10%, respectively.

The filtered high-quality reads were assembled *de novo* using SPAdes version 3.15.5 (11). The resulting contigs were further scaffolded using Geneious Prime v2025.2.1 with the reference genome of *Macrophomina phaseolina* (RefSeq accession GCA_020875535.1). Contigs and scaffolds shorter than 200 bp were removed from the assembly to improve assembly quality.

The final draft genome assembly consisted of 8,096 scaffolds with a total genome size of 50.3 Mb. The assembly had a scaffold *N*_50_ value of 27 kbp and an *L*_50_ of 523. The longest scaffold measured 176,496 bp. The GC content of the assembled genome was 52%, and the estimated sequencing depth was approximately 18×.

Genome completeness was assessed using BUSCO version 5.4.4 (12) with the ascomycota_odb10 lineage data set. The analysis revealed a completeness score of 96.3%, indicating that the genome assembly represents a highly complete fungal genome. Average nucleotide identity (ANI) analysis between the assembled genome and the reference genome of *M. phaseolina* showed 99.72% identity, confirming the species assignment.

The draft genome sequence of *Macrophomina phaseolina* isolate Karbala-1 has been deposited in the NCBI GenBank database under accession number JBVIJA000000000.1. This genome assembly expands the available genomic resources for the *M. phaseolina* species complex and provides a valuable reference for future studies aimed at understanding the genetic basis of pathogenicity, host adaptation, and epidemiology of this important soil-borne pathogen.

## Data Availability

The accession number of the internal transcribed spacer (ITS) region belonging to *M. phaseolina* isolate Karbala-1 is PZ118330.1. The genome data generated in this study are publicly available in the NCBI database. The project has been registered under BioProject accession number PRJNA1426132. The BioSample associated with the sequenced isolate, Karbala-1, is available under accession number SAMN55846687. The Whole-Genome Shotgun (WGS) assembly for this isolate has been deposited in DDBJ/ENA/GenBank under accession number JBVIJA000000000.1. The raw sequencing reads have been submitted to the NCBI Sequence Read Archive (SRA) under accession number SRR37297579. All associated records and data sets can be accessed through the NCBI database at https://www.ncbi.nlm.nih.gov.
